# Mysmenidae, a spider family newly recorded from Tibet (Arachnida, Araneae)

**DOI:** 10.3897/zookeys.549.6046

**Published:** 2016-01-05

**Authors:** Yucheng Lin, Shuqiang Li

**Affiliations:** 1 Key Laboratory of Bio-resources and Eco-environment (Ministry of Education), College of Life Sciences, Sichuan University, Chengdu, Sichuan 610064, China; 2 Institute of Zoology, Chinese Academy of Sciences, Beijing 100101, China

**Keywords:** Taxonomy, mysmenids, micro-orbweaver, new species, distribution

## Abstract

The spider family Mysmenidae is reported from Tibet for the first time. Two new species, *Chanea voluta* **sp. n.** (male and female) and *Mysmena lulanga* **sp. n.** (male and female) are found in eastern Tibet in high altitude. Morphological descriptions, diagnoses and comparative photos are provided for the two new species.

## Introduction

The eastern Tibet Plateau is one of the biodiversity hotspots in the world (Lei et al. 2015). The geographical location at the junction of different biogeographical realms, the wide range of habitats and climates along the extensive elevational range, the complex topography and the distinct geological history of this region have probably contributed to the evolution of an exceptionally species-rich and endemic-rich, specialized montane fauna. However, the lack of adequate research especially invertebrate results in multitudinous unknown new species to be discovered.

In a recent collection tour to eastern Tibet Plateau we yield a big number of spiders, including several new species. In this paper we described two new species of the family Mysmenidae. Mysmenidae includes 13 genera and 135 species worldwide (World Spider Catalog 2015). Of these mysmenid members elevation distribution drop from highest to lowest is nearly 3,300 meters. For example, *Tamasesia marplesi* was found at 3 meters off the coast of the jungle, in New Caledonia Island (Brignoli 1980). *Maymena roca* lives in the high mountain 3,300 meters above sea level, in Peru (Baert 1990). The new species described in this paper, i.e., *Chanea voluta* sp. n. collected from elevation between 2,140–3,060 meters and *Mysmena lulanga* sp. n. from elevation between 3,480–3,530 meters. The latter should be new highest record of elevation for spiders of the family Mysmenidae.

According to Li and Lin (2015) 4,282 species spider are recorded from China that belongs to 735 genera and 69 families. Of them, 37 mysmenid species of 8 genera (one Chinese mysmenid species, *Calodipoena cornigera*, is transferred to *Mysmena*, owing to *Mysmena* Simon, 1894 considered a senior synonym of *Calodipoena* Gertsch & Davis, 1936 by Lopardo and Hormiga 2015) are reported from Beijing, Chongqing, Guangxi, Guizhou, Hainan, Liaoning, Sichuan, Taiwan and Yunnan. The two new species described in this paper is the first record of the family Mysmenidae from Tibet.

## Material and methods

Specimens were examined and measured under a Leica M205 C stereomicroscope. Further details were studied under an Olympus BX43 compound microscope. Male palps and female genitalia were examined and photographed after they were dissected and detached from the spiders’ bodies. Vulvae were removed and treated in lactic acid before taking photos. To reveal the course of the spermatic duct, male palps were also treated in lactic acid and mounted in Hoyer’s Solution. All type specimens and preserved in 95% ethanol solution. Photos were taken with a Canon EOS 60D wide zoom digital camera (8.5 megapixels). The images were montaged using Helicon Focus 3.10.3 software (Khmelik et al. 2006).

All measurements are in millimeters, with leg measurements given in the following sequence: total length (femur, patella, tibia, metatarsus, and tarsus). The terminology mostly follows Lopardo et al. (2011) and Miller et al. (2009). The abbreviations used in text including: ARE – anterior eye row; ALE – anterior lateral eye; AME – anterior median eye; PRE – posterior eye row; PLE – posterior lateral eye; PME – posterior median eye. All specimens are deposited in the Institute of Zoology, Chinese Academy of Sciences (IZCAS) in Beijing.

## Taxonomy Family Mysmenidae Petrunkevitch, 1928

### Genus Chanea

Taxon classificationAnimaliaAraneaeMysmenidae

Miller, Griswold & Yin, 2009

Chanea Miller, Griswold & Yin, 2009: 54. Type species by original designation *Chanea suukyii* Miller, Griswold & Yin, 2009: 54.

#### Composition.

*Chanea suukyii* Miller, Griswold & Yin, 2009 and *Chanea voluta* sp. n.

#### Distribution.

China (Yunnan, Tibet)

#### Comments.

**Figure 1.**
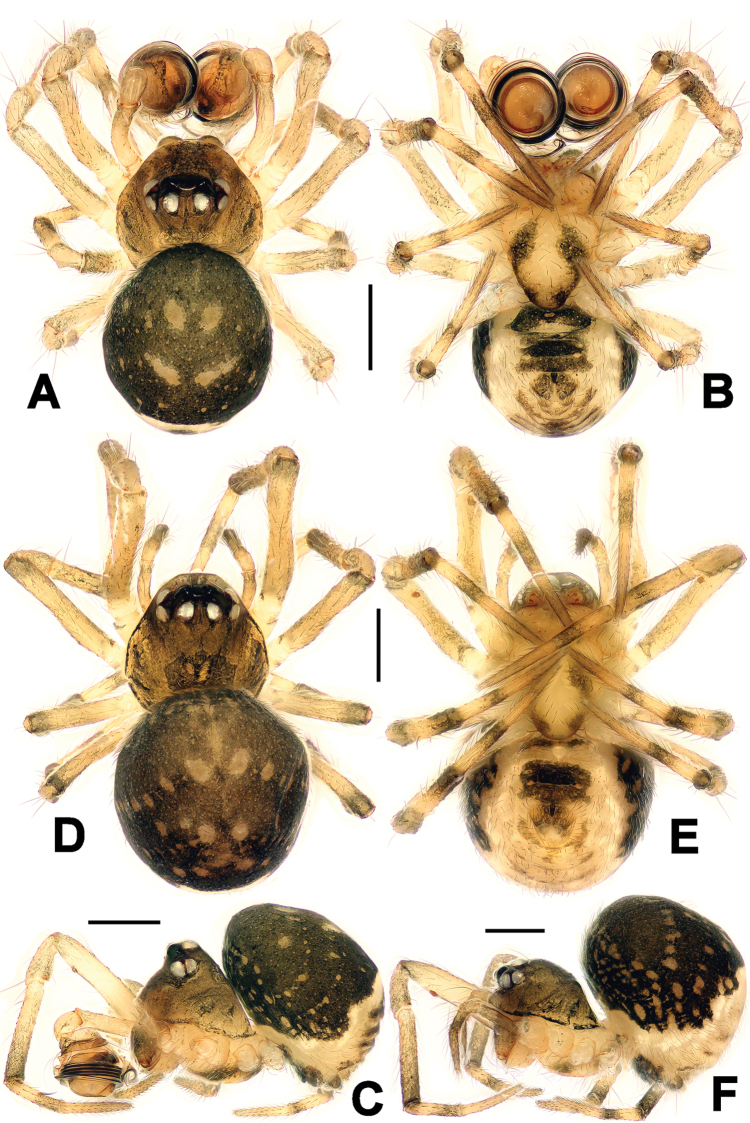
*Chanea voluta* sp. n., male holotype (**A–C**) and female paratype (**D–F**). **A, D** Habitus, dorsal **B, E** Habitus, ventral **C, F** Habitus, lateral. Scale bars = 0.20 mm.

**Figure 2.**
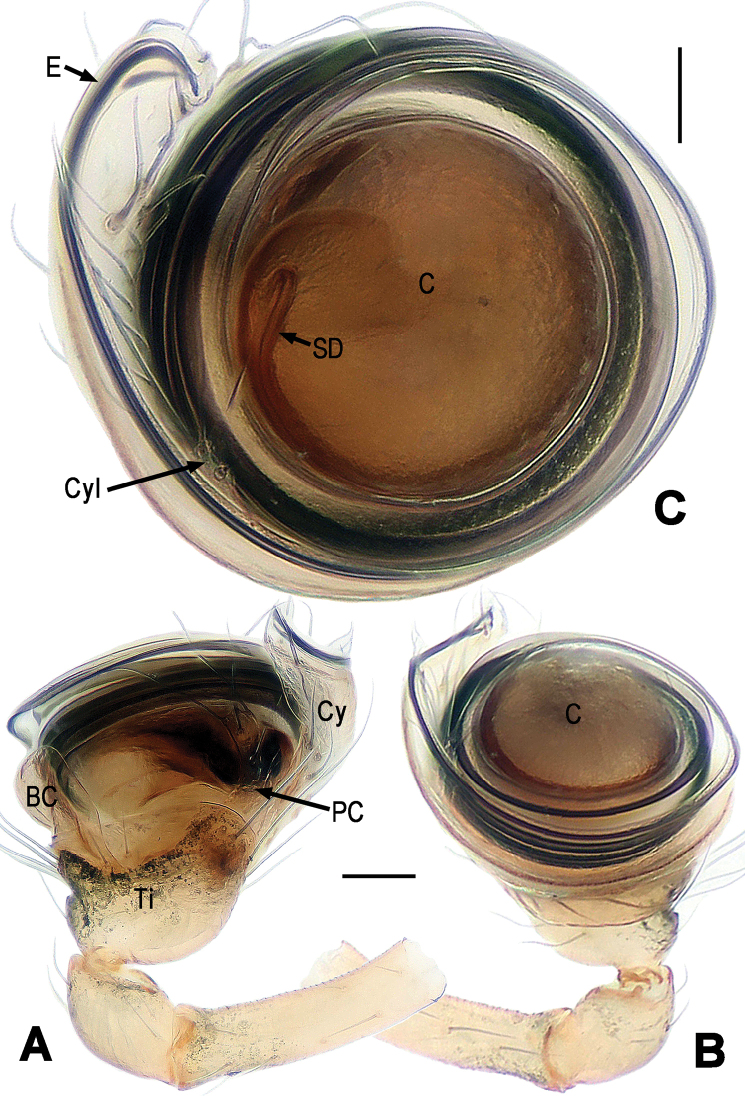
*Chanea voluta* sp. n., male holotype. **A** Left palp, prolateral **B** Left palp, retrolateral **C** Left palp, apical. Abbrs.: BC = base of cymbium; C = conductor; Cy = cymbium; Cyl = cymbial lobe; E = embolus; PC = paracymbium; SD = spermatic duct; Ti = tibia. Scale bars = 0.05 mm.

**Figure 3.**
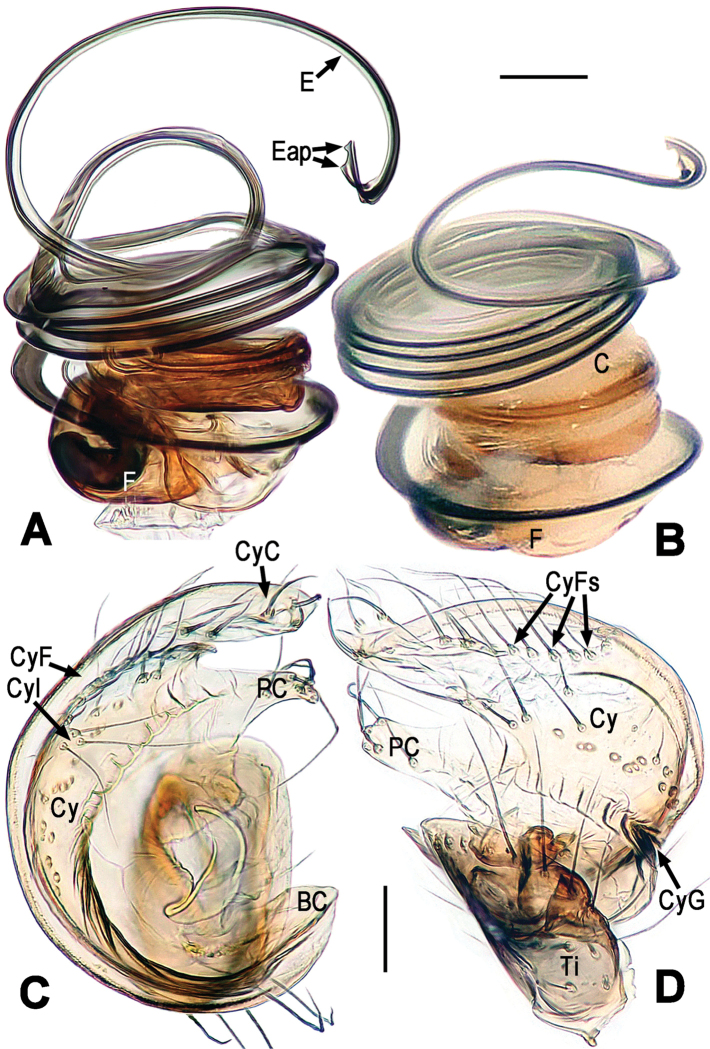
*Chanea voluta* sp. n., male holotype. **A, B** Palpal bulb, retrolateral **C** Cymbium, apical **D** Cymbium and palpal tibia, dorsal. **A, C–D** lactic acid-treated. Abbrs.: BC = base of cymbium; C = conductor; Cy = cymbium; CyC = cymbial conductor; CyF = cymbial fold; CyFs = setae on cymbial fold; CyG = cymbial groove; Cyl = cymbial lobe; E = embolus; Eap = embolar apophysis; F = fundus; PC = paracymbium; Ti = tibia. Scale bars = 0.05 mm.

**Figure 4.**
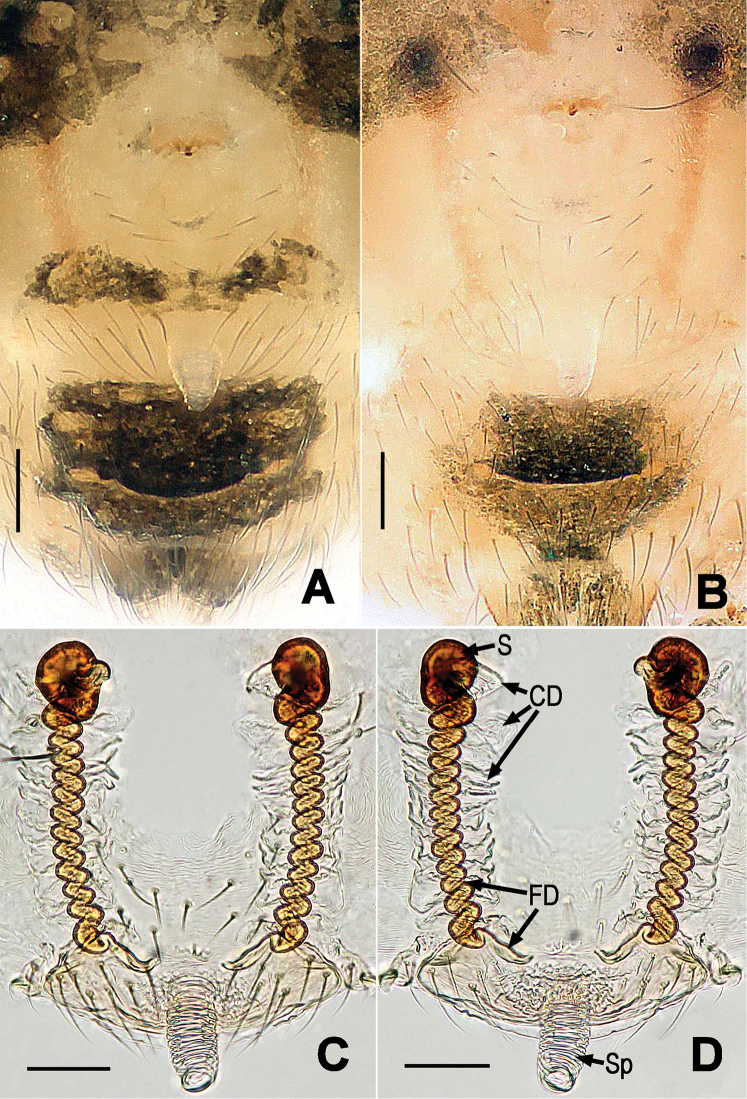
*Chanea voluta* sp. n., female paratype. **A** Epigynum, ventral **B** Epigynum (another paratype), ventral **C** Cleared vulva (lactic acid-treated), ventral **D** Epigynum, dorsal. Abbrs.: CD = copulatory duct; FD = fertilization duct; S = spermathecae; Sp = scape. Scale bars = 0.05 mm.

The genus *Chanea* was previously described in 2009 as monotypic (Miller et al. 2009). The type species, *Chanea suukyii*, was known only from the type locality in the Gaoligongshan Mountains, Yunnan Province, China. This spider species mainly live in leaf litter of the subtropical evergreen broadleaf forest. According to Miller et al. (2009), the diagnostic features of this genus differs from other mysmenids by the long embolus coiled into at least 5 loops encircles the conductor and subtegulum (figs 49A, 51B; Figs 2A–B, 3A–B), the entire distal part of the cymbium (fig. 49A; Fig. 3C–D), the widely spaced anterior median eyes (fig. 52B; Fig. 1A) and pair of macrosetae on the clypeus in male (fig. 52B), and the long copulatory ducts coiled around the fertilization ducts or coiled around fertilization ducts no less than 10 loops in female (fig. 49C; Fig. 4C–D). Miller et al. (2009) mentioned that the presence of a pair of clypeal marcosetae in male is also treated as one of the generic characters. But these are lacking from *Chanea voluta* sp. n. (Figs 1A, 1C). Therefore, we think that the extremely long, coiled embolus and the long, coiled copulatory ducts and/or fertilization ducts may be the main diagnoses for this genus. The paired macrosetae on the clypeus in male may just be an identifying character to this type species.

### Chanea voluta sp. n.

Taxon classificationAnimaliaAraneaeMysmenidae

http://zoobank.org/52473655-9A0D-46CC-9C8C-81BD373E7384

Figs 1 , 2 , 3 , 4 , 10

#### Type material.

*Holotype*: male (IZCAS), CHINA: Tibet Autonomous Region, Nyingchi Prefecture, Bomi County, the Road of Bomi to Medog, near the village of Baqiong (29°52.194'N, 95°43.505'E; Elevation: 2880 m), 19 July 2013, L.H. Lin & X.Z. Cao leg. *Paratypes*: 1 male and 9 females (IZCAS), same data as holotype; 1 male and 3 females (IZCAS), Nyingchi County, Bayi Town, Biri Holy Mt., Winding hill roads (29°51.334'N, 94°47.941'E; Elevation: 2900), 11 July 2013, L.H. Lin leg.; 5 males (IZCAS), Nyingchi Prefecture, the south of Mainling County (29°12.316'N, 94°12.649'E; Elevation: 3060 m), 13 August 2013, L.H. Lin leg.; 5 females (IZCAS), Nyingchi Perfecture, Bomi County, near Zhamo Town (29°50.859'N, 95°45.861'E; Elevation: 2800 m), 17 July 2013, L.H. Lin leg.; 2 males (IZCAS), Nyingchi Prefecture, 80 km of the road of Bomi to Medog (29°39.897'N, 95°29.963'E; Elevation: 2,140 m), 10 August 2013, X.Z. Cao leg., all types by manual sampling.

#### Etymology.

The specific name derived from the Latin word “volutus” = coiled, refers to the coiled embolus in male palp and the spiral fertilization duct in female vulva; adjective.

#### Diagnosis.

Male distinguished from *Chanea suukyii* Miller, Griswold & Yin, 2009 (see Miller et al. 2009: figs 49A–C, 50A–C, 51A–B, 52B–C) by the absence of paired macrosetea on the clypeus (Figs 1A, 1C), the longer paracymbium (Fig. 3C–D), the wider palpal bulb (Fig. 2A–C) and the variant embolic end (Fig. 3A–B), and female by the presence of a long scape (Fig. 4A–B), the larger spermatheca (Fig. 4C–D), the membranous fertilization ducts encircling around the coiled copulatory ducts (Fig. 4C–D).

#### Description.

**Male** (holotype). Somatic characters see Fig. 1A–C. Coloration: Prosoma brown centrally, dark marginally. Chelicerae somber. Sternum yellow, with two pair of dark speckles on shoulder and posterior. Opisthosoma black dorsally, yellow ventrally and posteriorly. Legs pale yellow, each tibia and metatarsus yellow proximally, black distally.

*Measurement*: Total length 0.69. Prosoma 0.35 long, 0.36 wide, 0.38 high. Opisthosoma 0.45 long, 0.41 wide, 0.50 high. Clypeus 0.13 high. Sternum 0.25 long, 0.24 wide. Length of legs: I 1.27 (0.44, 0.16, 0.27, 0.19, 0.21); II 1.04 (0.33, 0.14, 0.21, 0.17, 0.19); III 0.80 (0.24, 0.11, 0.14, 0.13, 0.18); IV 0.93 (0.30, 0.12, 0.18, 0.15, 0.18).

*Prosoma*: Carapace near round. Cephalic pars elevated, slope forward and backward. Clypeal margin concave. Ocular area at apex. Eight eyes in two rows. AME black, others white. AME smallest, ALE largest. ALE>PME>PLE>AME. ALE and PLE contiguous. ARE precurved, PRE straight. Chelicerae small, shorter slightly than endites (Fig. 1C). Endites with tiny serrula. Labium semiround, fused to sternum. Sternum triangular, plump.

*Legs*: formula: I-II-IV-III. Leg I with a subdistal-ventral sclerotized femoral spot and a prolateral-submesial metatarsal clasping macroseta. Patellae I–IV with a dorsal seta distally. Tibiae I–IV with a dorsal seta proximally. Tibiae I and II with 3 trichobothria, but 4 on tibia III and IV. Metatarsi I–IV with only one trichobothrium.

*Opisthosoma*: globular dorsally, triangular laterally. Spinnerets grey, the anteriors larger than the posteriors. Colulus small, black, finger-shaped. Anal tubercle pale.

*Palp* (Figs 2A–C, 3A–D): Large, strongly sclerotized. Femur normal. Patella short, with a few setae. Tibia swollen, wider than long, askew cup-shaped, covered with marginal long setae dorsally and ventrally (Figs 2A–B, 3D). Cymbium large, membranous, envelopes dorsal, retrolateral and ventral face of palpal bulb (Figs 2A, 3C–D). Base of cymbium broad (Figs 2A, 3C). Cymbial groove distinct, and rugose (Fig. 3D). Paracymbium developed, finger-shaped, with long seta distally (Fig. 3C–D). Internal margin of cymbium with an even row of setae on cymbial fold and a small cymbial lobe (Figs 2C, 3C). Conductor (or tegulum) smooth, compressed, rounded (Fig. 2B–C). Embolus very long, coiling into ca. 6 loops, tightly encircles conductor and subtegulum (Figs 2A–C, 3A–B). Embolar end slightly falcate, with tiny embolar apophysis (Fig. 3A–B), hidden behind distal cymbial conductor (Fig. 2A–B).

**Female** (one of paratypes). Somatic characters see Fig. 1D–F. Coloration: Same as in male.

*Measurement*: Total length 0.87. Prosoma 0.31 long, 0.38 wide, 0.36 high. Opisthosoma 0.48 long, 0.52 wide, 0.60 high. Clypeus 0.07 high, distinctly lower than in male. Sternum 0.27 long, 0.25 wide. Length of legs: I 1.40 (0.45, 0.19, 0.29, 0.22, 0.25); II 1.20 (0.39, 0.16, 0.24, 0.19, 0.22); III 0.87 (0.26, 0.12, 0.15, 0.15, 0.19); IV 1.10 (0.36, 0.13, 0.22, 0.18, 0.21).

*Prosoma*: Carapace near pear-shaped. Cephalic pars lower than in male. Eyes pattern, chelicerae, endites and sternum as in male.

*Legs*: Chaetotaxy and number of trichobothria same as in male, except for leg I without metatarsal clasping macroseta. Sclerotized femoral spot present at leg I and II. Leg formula: I-II-IV-III.

*Opisthosoma*: Globose dorsally. Genitalia black. Spinnerets grey, the anteriors larger than the posteriors. Colulus small, black, long finger-shaped.

*Vulva* (Fig. 4A–D): Epigynum weakly sclerotized, covered with short setae (Fig. 4A–B), with a membranous scape posterior-mesially (Fig. 4C). Scape blunt, rugose. Spermathecae small and egg-shaped, strongly sclerotized, set far anterior from epigastric furrow (Fig. 4B). Membranous copulatory ducts wrapped long, spiral fertilization ducts (Fig. 4C–D).

#### Distribution.

Known only from the type locality (Fig. 10).

### Genus Mysmena

Taxon classificationAnimaliaAraneaeMysmenidae

Simon, 1894

Mysmena Simon 1894: 558. Type species by original description *Theridion leucoplagiataum* Simon, 1879: 258 (= *Mysmena leucoplagiata* (Simon, 1879)).

#### Composition.

According to World Spider Catalog (2015), 27 described species, plus *Mysmena lulanga* sp. n. described here from Nyingchi Prefecture, Tibet, China.

#### Distribution.

Spain, Southern Europe to Azerbaijan, Saint Helena, Japan, Southwest China, Taiwan, Hainan Island, Vietnam, Oceania, islands of South Pacific, Guyana, Trinidad and Canada.

#### Comments.

The genus *Mysmena* was erected by Simon in 1894 initially as a genus of Theridiiae with the type species *Theridion leycoplagiatum* Simon, 1879; later transferred to Symphytognathidae by Forster (1959), and then to Mysmenidae from Symphytognathidae by Forster and Platnick (1977). In recent years, research on species description reports of this genus mainly comes from China (Miller et al. 2009; Lin and Li 2008, 2013a, 2013b), Japan (Ono 2010), Queensland (Lopardo and Michalik 2013) and Canada (Lopardo et al. 2008). Lopardo and Hormiga (2015) suggested that *Calodipoena*, *Itapua*, *Calomyspoena*, *Tamasesia*, and *Kekenboschiella* are synonymized with *Mysmena* basing on the results of phylogeny and evolutionary of the family Mysmenidae. Several optimized synapomrophies shared by most of this genus were proposed, include the spermatic duct switchback distally benting at a right angle, the presence of a long ventral scape, the weakly sclerotized fertilization ducts and the vulva with a distinguishable wall (Lopardo and Hormiga 2015).

### Mysmena lulanga sp. n.

Taxon classificationAnimaliaAraneaeMysmenidae

http://zoobank.org/D905C599-A81F-44E7-A7C8-7CD21C4F6277

**Figure 5.**
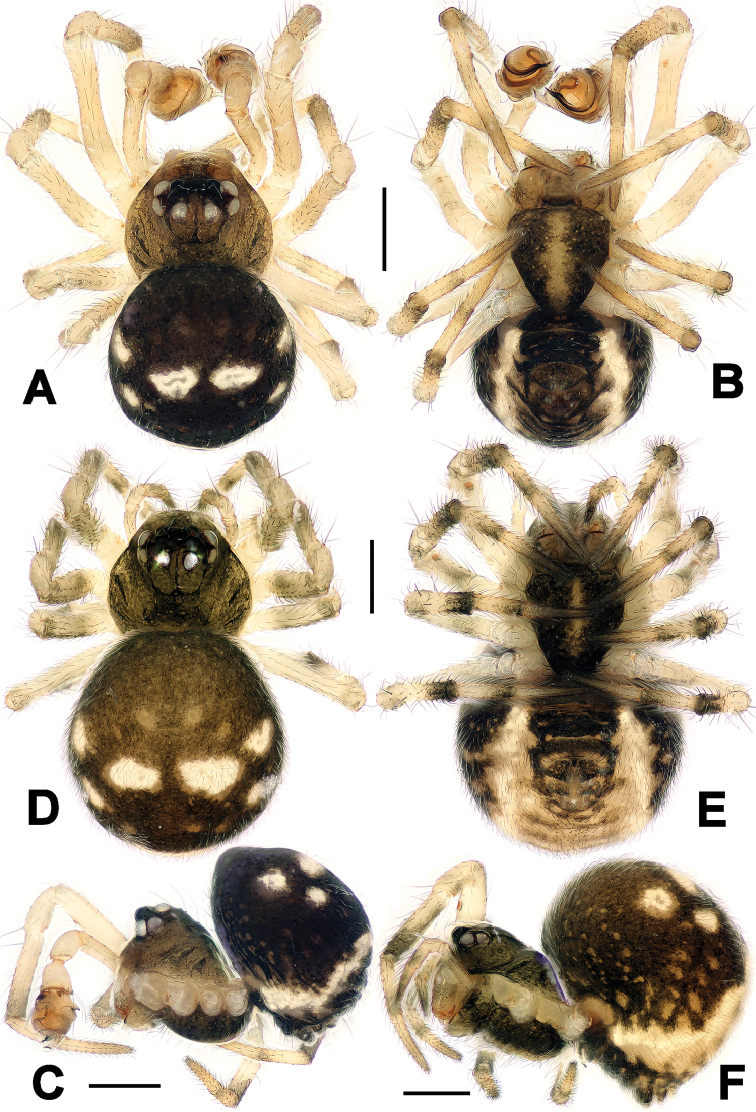
*Mysmena lulanga* sp. n., male holotype (**A–C**) and female paratype (**D–F**). **A, D** Habitus, dorsal **B, E** Habitus, ventral **C, F** Habitus, lateral. Scale bars = 0.20 mm.

**Figure 6.**
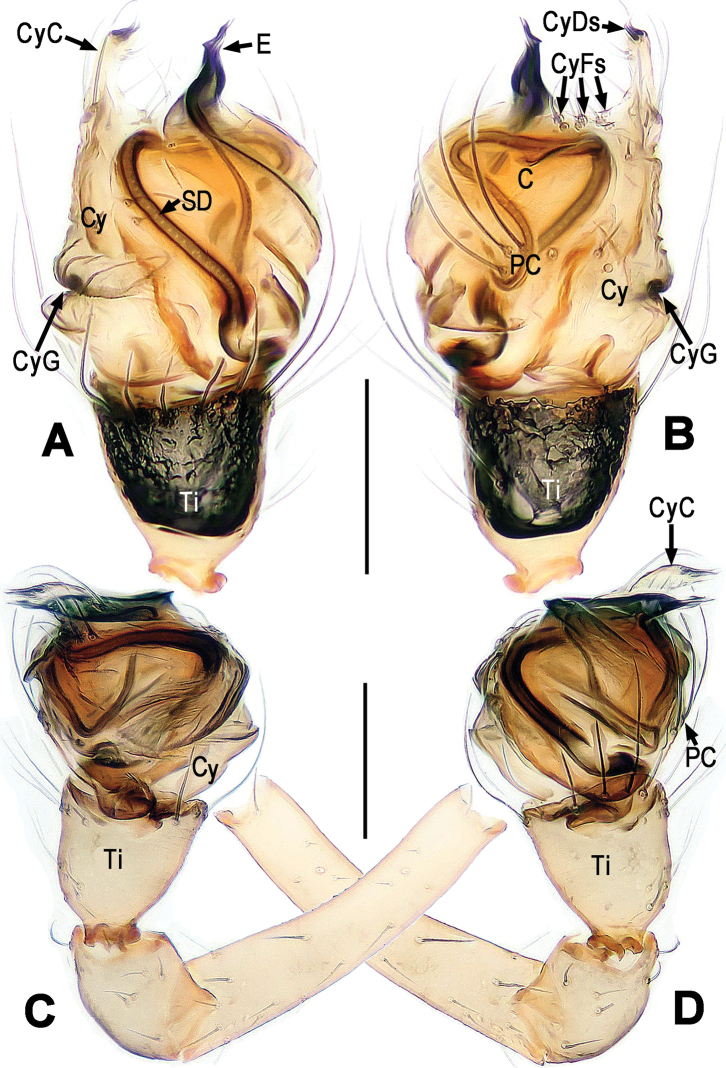
*Mysmena lulanga* sp. n., male holotype. **A** Left palp, ventral **B** Left palp, dorsal **C** Left palp, prolateral **D** Left palp, retrolateral. Abbrs.: C = conductor; Cy = cymbium; CyC = cymbial conductor; CyDs = cymbial distal macroseta; CyFs = setae on cymbial fold; CyG = cymbial groove; E = embolus; PC = paracymbium; SD = spermatic duct; Ti = tibia. Scale bars = 0.10 mm.

**Figure 7.**
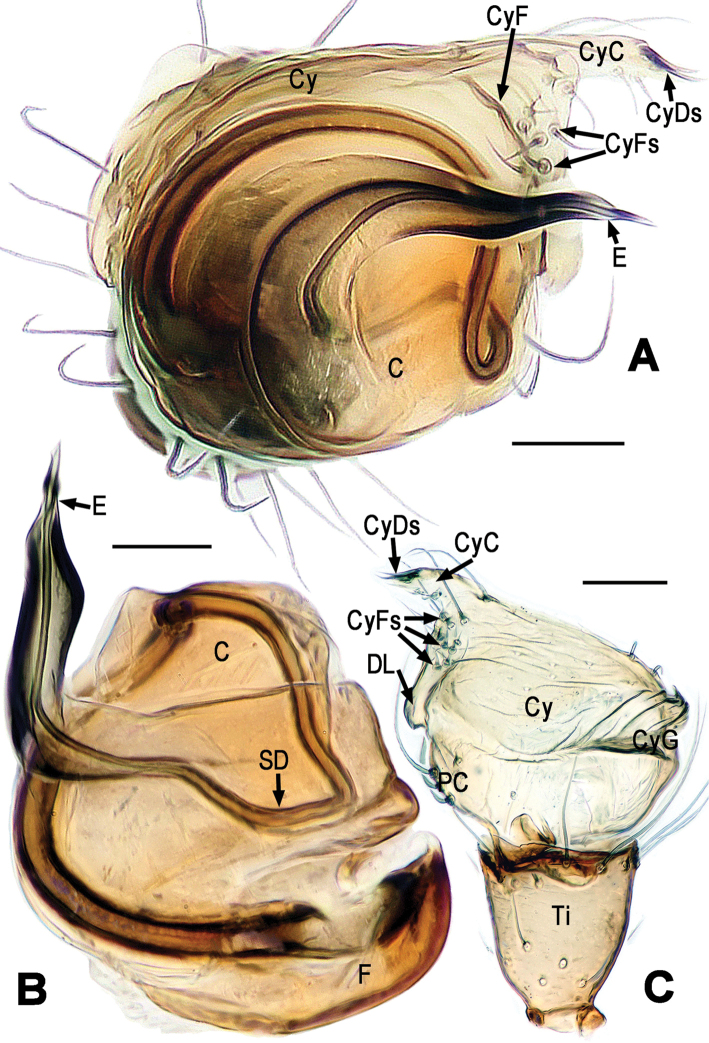
*Mysmena lulanga* sp. n., male holotype. **A** Left palp, apical **B** Palpal bulb, ventral-apical **C** Cymbium and palpal tibia, prolateral. **A–C** lactic acid-treated. Abbrs.: C = conductor; Cy = cymbium; CyC = cymbial conductor; CyDs = cymbial distal macroseta; CyF = cymbial fold; CyFs = setae on cymbial fold; CyG = cymbial groove; DL = distal lobe of cymbium; E = embolus; PC = paracymbium; SD = spermatic duct; Ti = tibia. Scale bars = 0.05 mm.

**Figure 8.**
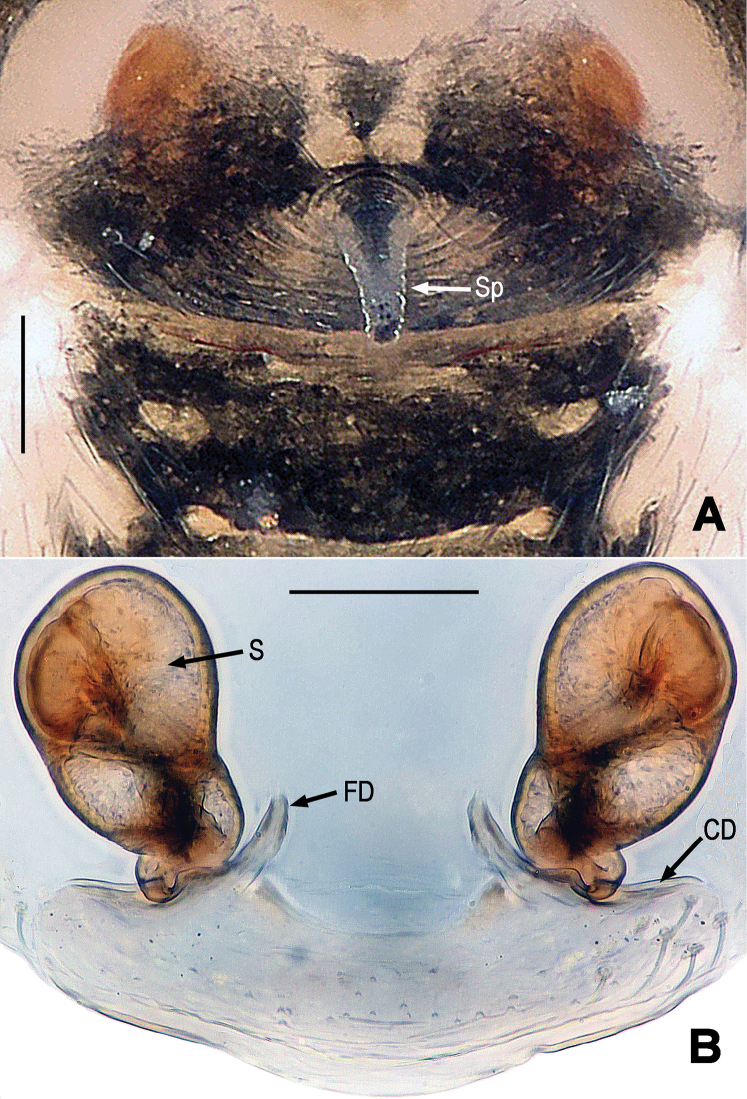
*Mysmena lulanga* sp. n., female paratype. **A** Epigynum, ventral **B** Cleared vulva (lactic acid-treated, omitted scape), dorsal. Abbrs.: CD = copulatory duct; FD = fertilization duct; S = spermathecae; Sp = scape. Scale bars = 0.05 mm.

**Figure 9.**
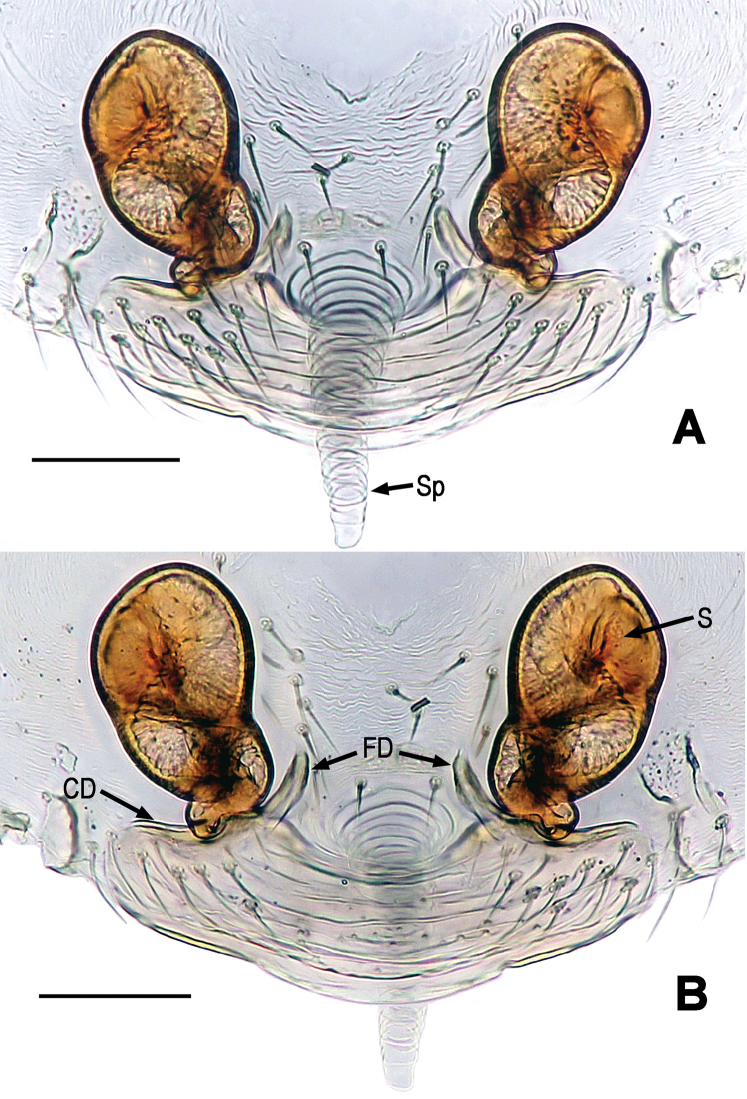
*Mysmena lulanga* sp. n., female paratype. **A** Epigynum (lactic acid-treated), ventral **B** Cleared vulva (lactic acid-treated), dorsal. Abbrs.: CD = copulatory duct; FD = fertilization duct; S = spermathecae; Sp = scape. Scale bars = 0.05 mm.

**Figure 10.**
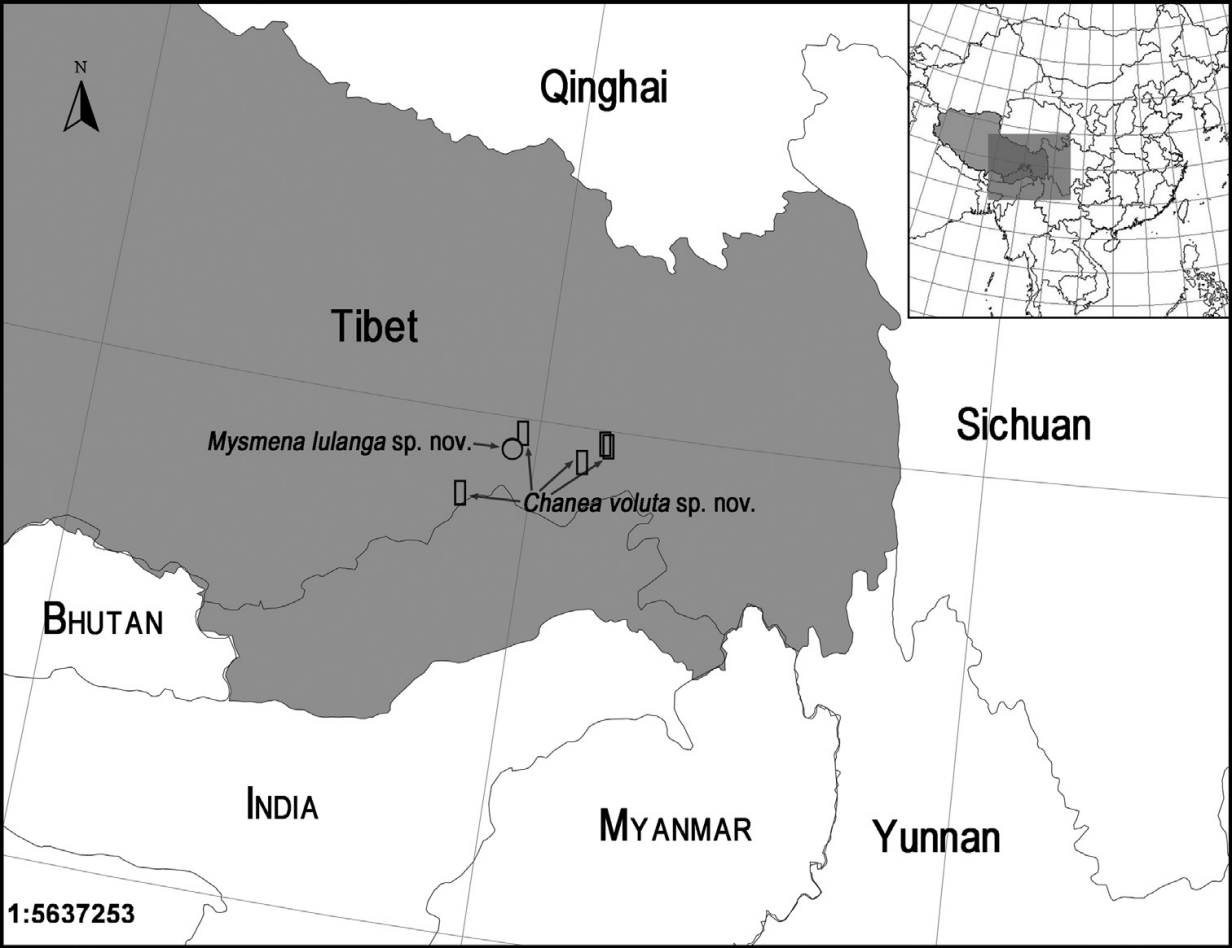
Records of two new species of Mysmenidae from Tibet, China.

Figs 5 , 6 , 7 , 8 , 9 , 10

#### Type material.

*Holotype*: male (IZCAS), CHINA: Tibet Autonomous Region, Nyingchi County, the east of Lulang Town (29°41.984'N, 94°43.657'E; Elevation: 3480 m), 14 July 2013, L.H. Lin leg. *Paratypes*: 1 male and 7 females (IZCAS), same data as holotype; 1 male and 10 females (IZCAS), Nyingchi County, the east of Lulang Town (29°41.449'N, 94°43.605'E; Elevation: 3530 m), 14 July 2013, L.H. Lin leg., all types by manual sampling.

#### Etymology.

The specific name derives from the type locality. The epithet is a noun in apposition.

#### Diagnosis.

Male distinguished by the cymbial conductor with two distal macrometae (Figs 6A–B, 7C). Female distinguished from other congeners by the ovate spermatheca and the vulva without membranous copulatory duct or/and fertilization duct (Figs 8B, 9A–B). Compared with other Chinese *Mysmena* species, the new species and *Mysmena baoxingensis* Lin & Li, 2013 have the most similar in configuration of palp and inner form of epigynum (see Lin and Li 2013a: figs 14A–E, 15A–D), but male differs from the latter by the shorter, wider embolus (Figs 6A–B, 7A–B), the two cymbial distal macrosetae (Figs 6B, 7A, 7C), and female by the near egg-shaped spermatheca (Figs 8B, 9A–B), the upswept fertilization ducts (Figs 8B, 9B) and the tapering, non-sclerotized scape (Figs 8A, 9A). Distinguished from the Vietnamese species *Mysmena maculosa* and *Mysmena tamdaoensis* (Lin & Li, 2014) by the lack of cymbial spur and the female abdomen without posterior projection, or by a simple embolus and the epigynum with a long scape. Further distinguished from other *Mysmena* species in Sulawesi (Baert 1988), New Guinea (Baert 1982, 1984; Forster 1959), Samoa (Marples 1955), North America (Lopardo and Dupérré 2008) and Latin America (e.g. Baert and Maelfait 1983; Gertsch 1960; Gertsch and Davis 1936; Levi 1956) by the shorter embolus and the lack of membranous copulatory duct (Figs 6A–B, 9B).

#### Description.

**Male** (holotype). Somatic characters see Fig. 5A–C. Coloration: Prosoma darkish, ocular area black. Sternum black, with a pale longitudinal stripe centrally. Opisthosoma black dorsally, with three pair of white speckles, one large centrally and two small marginally, white pigment stripe at the lateral and posterior, black ventrally. Femora of legs pale yellow, other segments pale proximally, darkish distally.

*Measurement*: Total length 0.71. Prosoma 0.33 long, 0.35 wide, 0.31 high. Opisthosoma 0.45 long, 0.43 wide, 0.50 high. Clypeus 0.09 high. Sternum 0.24 long, 0.25 wide. Length of legs: I 1.18 (0.38, 0.16, 0.24, 0.18, 0.22); II 1.04 (0.32, 0.14, 0.22, 0.16, 0.20); III 0.79 (0.23, 0.12, 0.14, 0.13, 0.17); IV 0.94 (0.29, 0.13, 0.18, 0.15, 0.19).

*Prosoma*: Carapace near round. Cephalic pars elevated, sharply vertical forward and slope backward. Clypeal margin concave. Ocular area at apex. Eight eyes in two rows. AME black, others white. ALE and PLE contiguous. AME smallest, ALE equal to PME in size. ALE=PME>PLE>AME. ARE slightly precurved, PRE slightly recurved. Chelicerae pale, small, shorter than endites (Fig. 5C). Endites with tiny serrula. Labium rectangular, wider than long, fused to sternum. Sternum cordiform, plump.

*Legs*: formula: I-II-IV-III. Leg I with a prolateral-mesial metatarsal clasping macroseta. Sclerotized femoral spot present at leg I and II. Patellae I–IV with a dorsal seta distally. Tibiae I–IV with a dorsal seta proximally. Tibiae I and II with 3 trichobothria, but 4 on tibia III and IV. Metatarsi I–IV with only one trichobothrium.

*Opisthosoma*: Globular dorsally. Spinnerets dark, the anteriors larger than the posteriors. Colulus tiny, black. Anal tubercle darkish.

*Palp* (Figs 6A–D, 7A–C): Femur long, ca. 3 times as long patella. Patella short, with a few setae. Tibia swollen, cup-shaped, covered with long setae on distal margin ventrally and dorsally (Fig. 6A–D). Cymbium membranous, wide, arisen from tibial margin prolaterally (Figs 6C, 7D). Cymbial groove distinct, rugose (Figs 6A–B, 7C). Paracymbium small semiround, undevolped (Fig. 7C). Distal lobe of cymbium auriform (Fig. 7C). Setae on cymbial fold irregular arrange (Figs 6B, 7A, 7C). Cymbial conductor horn-shaped, with two strong cymbial distal marocsetae at apex (Figs 6B, 7A). Conductor (or tegulum) smooth, globular (Figs 6B, 7A–B). Spermatic duct visible through subtegulum (Figs 6A–B, 7A–B). Embolus wide, strongly sclerotized. Embolar end sharp (Fig. 7A–B).

#### Female

(one of paratypes). Somatic characters see Fig. 5D–F. Coloration: Same as in male.

*Measurement*: Total length 0.95. Prosoma 0.38 long, 0.40 wide, 0.35 high. Opisthosoma 0.66 long, 0.60 wide, 0.67 high. Clypeus 0.08 high, slightly lower than in male. Sternum 0.26 long, 0.27 wide. Length of legs: I 1.25 (0.41, 0.17, 0.26, 0.19, 0.22); II 1.10 (0.35, 0.16, 0.22, 0.17, 0.20); III 0.87 (0.26, 0.14, 0.15, 0.14, 0.18); IV 1.04 (0.33, 0.15, 0.21, 0.16, 0.19).

*Prosoma*: Carapace near pear-shaped. Cephalic pars lower than in male. Eyes pattern, chelicerae, endites and sternum as in male.

*Legs*: Chaetotaxy and number of trichobothria same as in male, except for leg I without metatarsal clasping macroseta. Sclerotized femoral spot present at leg I and II. Leg formula: I-II-IV-III.

*Opisthosoma*: Globose dorsally. Spinnerets grey, the anteriors larger than the posteriors. Colulus small, black, tongue-shaped.

*Vulva* (Figs 8A–B, 9A–B): Epigynum large, weakly sclerotized. Epigynal area covered with short setae (Fig. 8A). A long, tapering scape arising from the middle position between spermathecae, not from epigynal posteromargin mesially (Figs 8A, 9A). Spermathecae large, strongly sclerotized, near egg-shaped (Figs 8B, 9B). A translucent, broad anterior genital plate lain beneath spermathecae (Figs 8B, 9B). Copulatory ducts short, derives from ventral-posterior position of spermathecae ventrally, and connected with anterior corner of genital plate (Figs 8B, 9A–B). Fertilization ducts short, upswept, connected with dorsal-posterior position of spermathecae (Fig. 8B).

#### Distribution.

Known only from the type locality (Fig. 10).

## Discussion

Finding the species *Chanea voluta* sp. n. allowed us to clearly place the genus *Chanea* within the Mysmenidae, by the presence of the most important characters of the genus that the type species *Chanea suukyii* and *Chanea voluta* sp. n. both share: an extra-long (at least five loops) coiled embolus, and the very long (at least ten loops) spiral copulatory duct or/and fertilization duct. In addition, another significant common feature between them is the relatively small spermatheca located far from the epigastric groove. These common features indicate that these species belong to the same genus. As for the clypeal setae in male, although quite typical for *Chanea suukyii*, we think this may be only a species specific character. Like the front cheliceral setae found in the males of some *Mysmena* species, some species have them (e.g. *Mysmena arcilonga*, *Mysmena rostella*, *Mysmena vangoethemi*, *Mysmena taiwanica*), but others do not. This same situation also appears in the genus *Gaoligonga*. The scape may be present or absent; the same is true in other mysmenid species. However, these characters are still in doubt.

In conclusion, the monophyly and circumscription of the genus *Chanea* and its relationships within Mysmenidae needs more study (Lopardo and Hormiga 2015).

## Supplementary Material

XML Treatment for Chanea

XML Treatment for Chanea voluta

XML Treatment for Mysmena

XML Treatment for Mysmena lulanga
